# Interactive Clustered Heat Map Builder: An easy web-based tool for creating sophisticated clustered heat maps

**DOI:** 10.12688/f1000research.20590.2

**Published:** 2020-03-19

**Authors:** Michael C. Ryan, Mark Stucky, Chris Wakefield, James M. Melott, Rehan Akbani, John N. Weinstein, Bradley M. Broom

**Affiliations:** 1 In Silico Solutions, Fairfax, VA, 22031, USA; 2 Department of Bioinformatics and Computational Biology, University of Texas MD Anderson Cancer Center, Houston, TX, USA; 3 Department of Systems Biology, University of Texas MD Anderson Cancer Center, Houston, TX, USA

**Keywords:** Bioinformatics, Genomics, Heat Map, Web Tool, Website, Hierarchical Clustering

## Abstract

Clustered heat maps are the most frequently used graphics for visualization and interpretation of genome-scale molecular profiling data in biology.  Construction of a heat map generally requires the assistance of a biostatistician or bioinformatics analyst capable of working in R or a similar programming language to transform the study data, perform hierarchical clustering, and generate the heat map.  Our web-based Interactive Heat Map Builder can be used by investigators with no bioinformatics experience to generate high-caliber, publication quality maps.  Preparation of the data and construction of a heat map is rarely a simple linear process.  Our tool allows a user to move back and forth iteratively through the various stages of map generation to try different options and approaches.  Finally, the heat map the builder creates is available in several forms, including an interactive Next-Generation Clustered Heat Map that can be explored dynamically to investigate the results more fully.

## Introduction

Many thousands of publications on genomics studies include clustered heat maps (CHMs) because the hierarchical clustering and intuitive visualization provide insight into the relationships among sample sub-groups and key biological processes ^1– 8^. Construction of a CHM requires data transformation, application of clustering methods, association of covariate (classification) data, and production of the heat map visualization. Generally, those tasks require the assistance of an analyst with biostatistics or bioinformatics skills who can work in R or a similar language to manipulate the study data and generate the map. This is usually not a simple linear process because data transformation and clustering methods are often revisited to find the ideal match for the study, and modifications are often made to heat map visualizations to select the best colors, adjust covariates, insert gaps, etc. Our Interactive CHM Builder is a web-based tool for data transformation, clustering, and generation of high-quality heat maps. It can be used by investigators with no bioinformatics experience and only modest exposure to biostatistical methods. The tool guides users through the steps of creating a heat map and supports iterative refinement of the map by working backward and forward through the steps to refine data transformation, annotation, clustering, and formatting options. (Caveat: Iterative exploration of different options may introduce a multiple-comparisons issue that would have to be taken into account if the map were used for formal statistical inference, rather than discovery.)

One obvious limitation of traditional heat maps is that they contain a huge amount of information but are static in nature and do not readily support a deeper exploration of the biology behind the image. The Interactive CHM Builder produces traditional heat map images as PDF files but can also produce interactive next-generation CHMs (NG-CHMs). NG-CHMs support interactive exploration of patterns in the data through zooming, panning, searching, and advanced link-outs to dozens of external resources. An NG-CHM file can be downloaded and viewed locally with the NG-CHM viewer and, importantly, can be embedded in a study results webpage or publication.

The Interactive CHM Builder ^9^, available at https://build.ngchm.net/NGCHM-web-builder/, is easy to try out using sample data provided at the site. Other methods of producing NG-CHMs, including an R library and a set of tools for the Galaxy platform ^10, 11^, are described at https://www.ngchm.net/.

## Methods

### Implementation

The Interactive Builder ^9^ is web-based application that accepts an uploaded data matrix and then walks the user through several steps to transform the data, perform hierarchical clustering, and format the resulting CHM. The application is implemented as HTML, CSS, and JavaScript on the browser-side and Java servlets on the web server. Data manipulation and heat map generation are implemented in Java classes used by the servlets. The clustering is performed by a servlet using the Renjin engine ( https://www.renjin.org) to perform R clustering functions in Java. Browser sessions are tracked by the server to create a working area for each user and prevent users from seeing each other’s data or maps. In addition to the working version of the data matrix on which transformations are performed, an original version of the matrix is preserved. Returning to a previous matrix state is accomplished by restoring the original version and then re-applying transformations until the requested state is restored. The site retains constructed heat maps and the related uploaded data only for the duration of the HTTP session.

**Figure 1.**
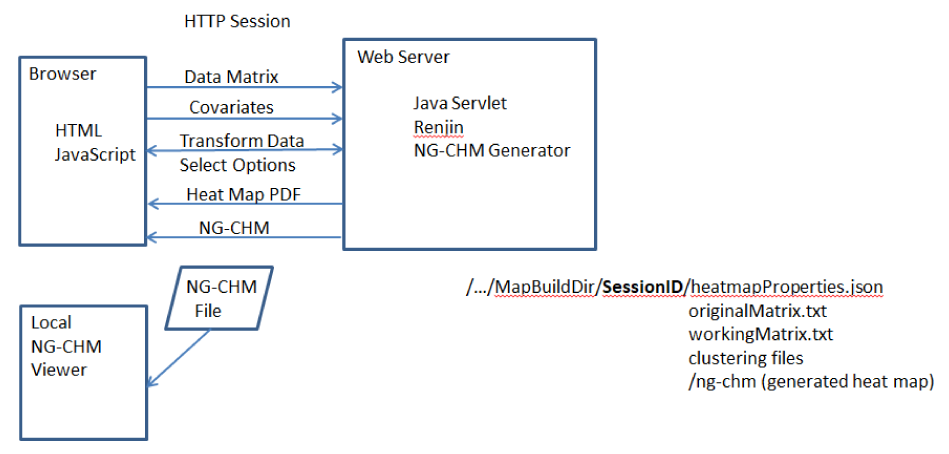
High-level overview of the interaction of heat map builder components. Heat maps are built on a webserver. A browser session ID is used to create a separate, temporary working area for each user. Heat map construction sessions are cleaned up when the session is ended, but PDF and NG-CHM heat map files can be downloaded.

A Java NG-CHM heat map generator .jar file is used to construct the heat map repeatedly as options are selected in each step of the builder. The heatmapProperties.json file, which contains all options selected by the user, conveys the selected options to the generator. The current NG-CHM file set is stored in a directory under the session ID. The NG-CHM file is a zipped version of the NG-CHM directory. The downloaded .ngchm file can be saved locally and viewed interactively using a local instance of the NG-CHM viewer that can also be downloaded from the builder site. An overview is given in Figure 1.

The full source code for the Interactive Builder is available in GitHub.

### Operation

There is no need to install software to use the Interactive Builder ^9^ it is available for public use on our server at https://build.ngchm.net/NGCHM-web-builder/. If, however, a local private installation of Interactive Builder is preferred, there are two simple installation methods.

Organizations familiar with Docker can run the Builder as a Docker container ( https://docs.docker.com/). To do this, clone the git repository. The base folder of this repository has a docker build file. Run the docker build command in this directory with a –t option to name the resulting docker image. For example: docker build . –t nghm_builder. Then use the docker run command to start a container using the image. The heat maps created by the software are transient and last only for the duration of a user http session so there is no need to mount an external directory to the container for persistent storage. The port for connecting to the webserver in the container does need to be specified in the docker run command. Connect the desired external port to the tomcat instance in the container. For example, docker run --name=“ngchm_builder” -d -p 8888:80 ngchm_builder. Users should then be able to connect to Interactive Builder using their browser and the URL of the docker container. For example, http://<docker machine IP or URL>/ NGCHM-web-builder.

The other option for deploying the software is to install it on an existing web server like tomcat ( https://tomcat.apache.org/tomcat-9.0-doc). To do this, first clone the git repository and then use the ant script, ant_buildfile.xml in the NG-CHM_GUI_BUILDER folder to create a .war file. Then simply copy the .war file to the webapps directory of the web server. The application should then be available at http://<server URL>/ NGCHM-web-builder.

## Use case

The starting point for a CHM is a matrix of data. In this use-case example, we focus on gene expression data from The Cancer Genome Atlas (TCGA) bladder cancer project ^12, 13^. The rows and columns of the matrix require identifiers, in this case sample ids and gene symbols, and the cells of the matrix must be numeric values. The builder will accept either a tab-delimited text file (*.txt), comma-separated text file (*.csv) or Excel spreadsheet (*.xlsx). 

### Select matrix

**Figure 2.**
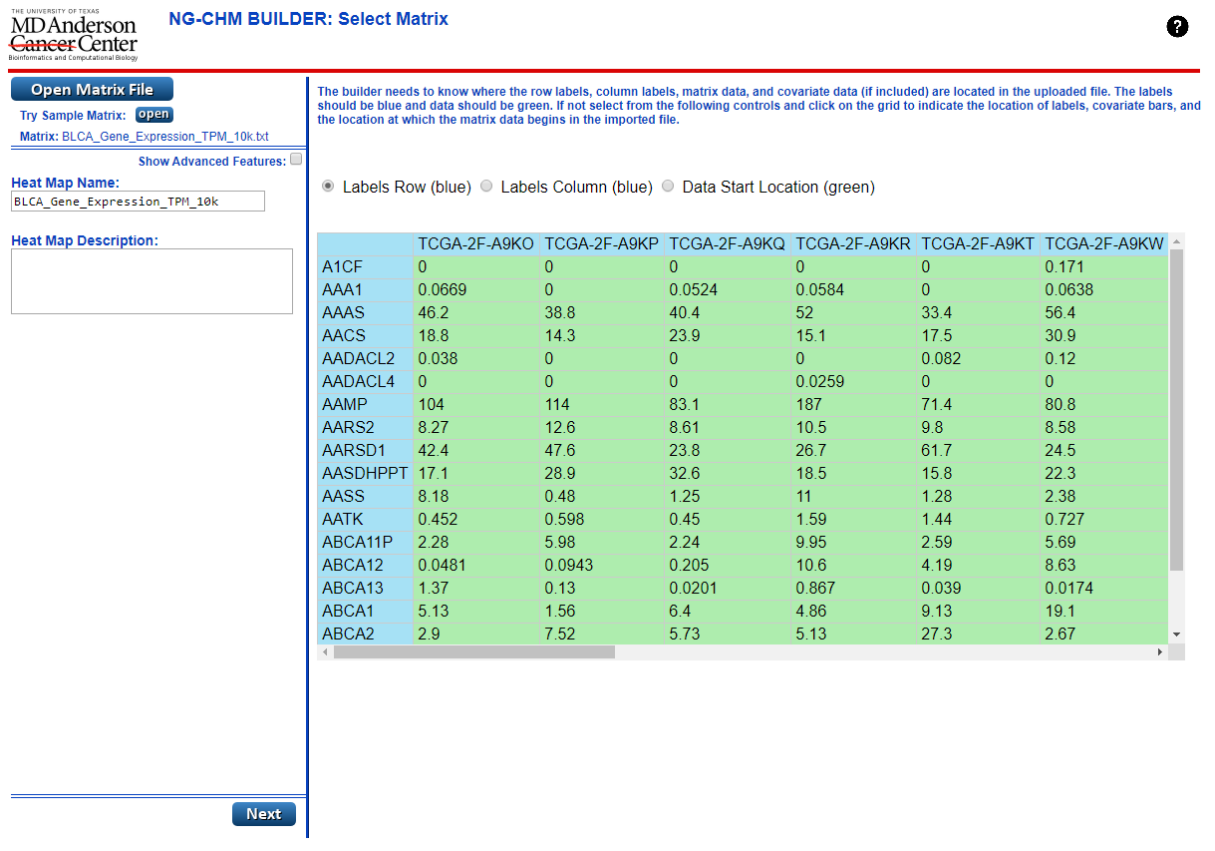
Heat map creation starts with importing a text matrix file (e.g., *.txt, *.csv or Excel *.xlsx file) and identifying the row labels, column labels and numerical data values.

 The Open Matrix File button on the first page of the builder ( Figure 2) is used to upload the data matrix. A name and optional description to be associated with the heat map are entered. When the data have been loaded, the Select Matrix page will show the first few rows and columns of the matrix. It is important that the builder correctly identify the row labels, column labels, and matrix data; the backgrounds of labels and matrix data should be blue and green, respectively. If the input file has extra rows or columns, you may need to correct the identification of labels and matrix data by selecting the appropriate radio button and then clicking on the correct location in the matrix displayed.

Note that several screens in the builder include advanced features that are hidden by default to simplify the process for first-time users. The use-case example here does not require advanced features, but be aware that additional capabilities can be accessed using the Advanced Features checkbox.

### Transform/filter the data

**Figure 3.**
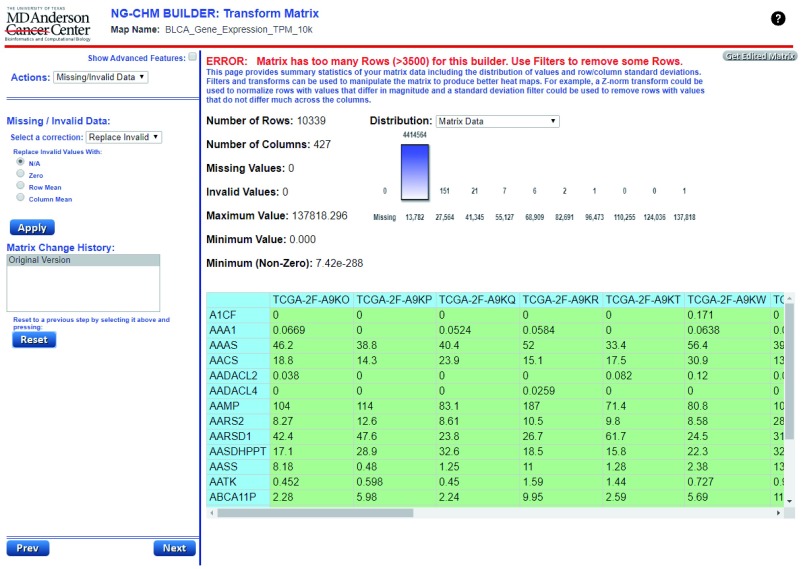
The data transform page makes it easy to perform operations on the matrix like log transformation or filtering to reduce and normalize data.

Creating a good heat map depends on proper data preparation. The second step in the build process is the Data Transform page ( Figure 3), which provides three primary categories of matrix transformations: functions that identify and replace missing/invalid values, filters to remove rows or columns, and transforms to perform mathematical operations on data values. There are additional choices in advanced mode for transposing the matrix and calculating correlations. 

The right-hand panel of the Transform page provides summary statistics about the data matrix, including the number of rows and columns, a histogram of the data distribution, and an indication of the number of invalid cells in the matrix. The top of the page also provides suggestions about transformations that can be performed and flags any problems with the data. The use-case matrix is too large for the Interactive Builder to use in creating a heat map interactively; the clustering time, which increases approximately as the square of the larger matrix dimension for most clustering algorithms, is limiting. Currently, the website limits the heat map to no more than 5,000 total rows and columns (for example 1,000 samples and 4,000 genes) at the clustering stage. However, users can upload much larger matrices as long as filters on the transform page reduce the size to 5,000. For practical purposes, that often means extracting the most relevant data (e.g., with few enough missing values, sufficient signal, and sufficient standard deviation across samples) for clustering. We are also progressively increasing the size limit as compute power and clustering algorithms advance.

For this use case the transform tab is used to fix duplicate column headers; set a minimum threshold to reduce the influence of noise in the heat map; normalize the data with a log transform and mean center; and filter to remove rows with many missing values and to keep only rows with strong variation across samples. The transforms applied were:

- Action: Duplicates Duplicates process: Rename. Column. Suffix duplicates with underscore and instance number. Apply.
- Action: Transform Data Transform: Threshold. Set Values Below 0.00001 to NA. Apply.
- Action: Transform Data Transform: Logarithmic. Log Base 10. Apply.
- Action: Transform Data Transform: Mean Center Row. Apply.
- Action: Filter Data Filter: Missing Data Row. Remove if > 50% Missing Values. Apply.
- Action: Filter Data Filter: Standard Deviation Row. Keep 500 rows with highest Standard Deviation. Apply.

**Figure 4.**
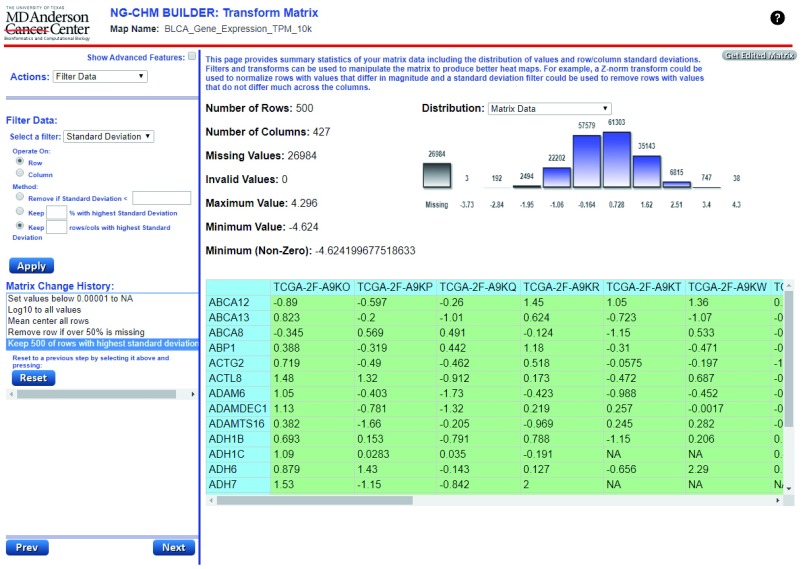
The transformed dataset has a better distribution and size for heat map generation than did the original. The history of transformations in the left-hand panel can be used to undo changes and revert to previous matrix states.

After applying the transformations, the matrix contains no errors and should be suitable for heat map generation ( Figure 4). Note that the left-hand panel shows the history of transformations performed on the matrix, and one can ‘undo’ back to any previous state of the matrix (including the original version) by clicking the desired previous state and hitting reset. More generally, the entire process of creating a heat map is iterative; the Next and Previous buttons can be used to return to previous steps to try different options. If, after generating the heat map, it appears that there should be more or fewer rows or different transforms, one can return to the pertinent screen and use the history and Reset option to adjust the data matrix. Finally, as an added feature, the Transform screen enables the user to download the filtered, transformed matrix for use in other analyses.

### Clustering

**Figure 5.**
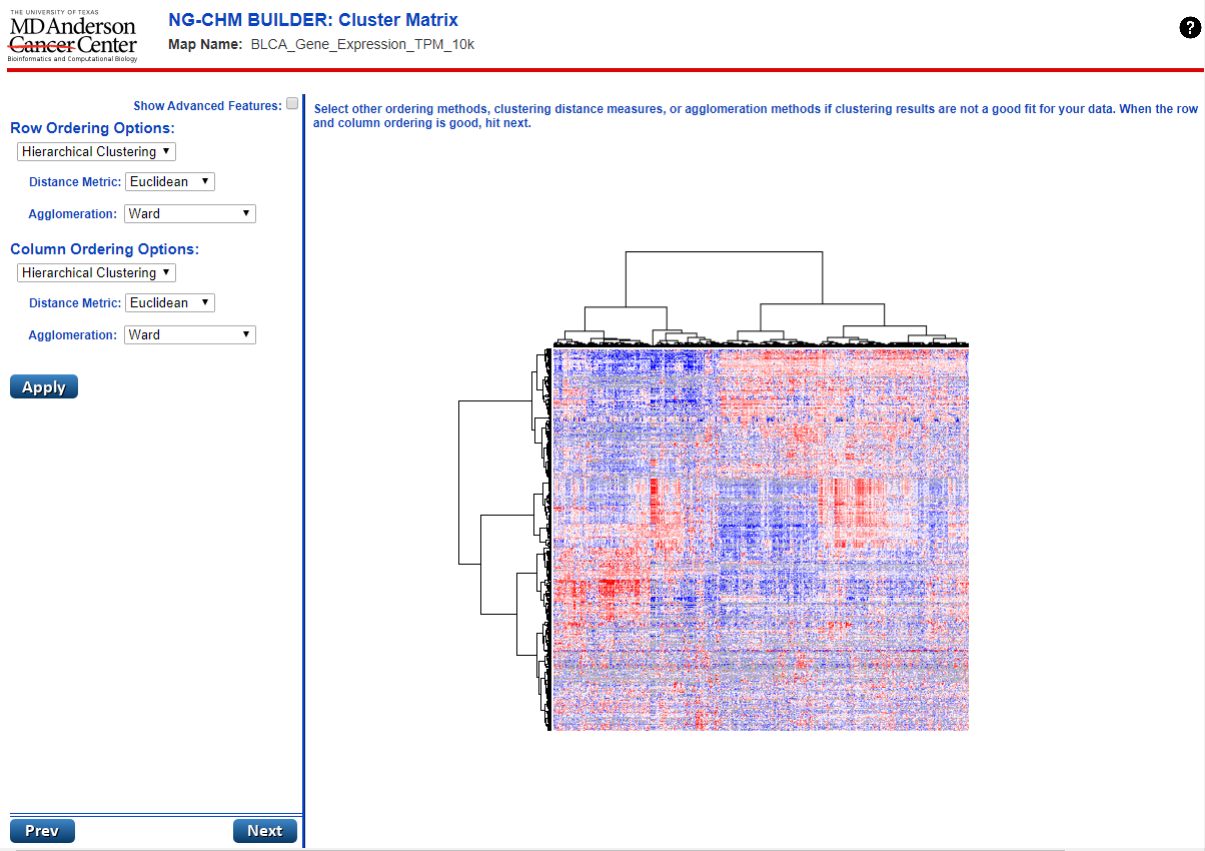
The clustering step supports many different clustering methods and distance measures. The Apply button performs clustering and displays the resulting dendrograms.

The next step is clustering ( Figure 5). The row order and column order drop-down menus can be used to select the clustering algorithm and distance measure to be applied to the rows and/or columns. Ward’s algorithm with Euclidean distance metric is one common choice, but the menus include many other possibilities, appropriate for different purposes and data characteristics. For the sample case, the Ward/Euclidean options provide strong separation in the dendrogram and interesting groups of samples. The menus also allow the rows and columns to be left in original order or randomized. Additional options will be provided in the future.

Please be aware that clustering of larger matrices may take a few minutes to complete. (The time it takes to cluster data increases approximately as the square of the number of rows or number of columns, whichever is larger.)

### Covariate bars

**Figure 6.**
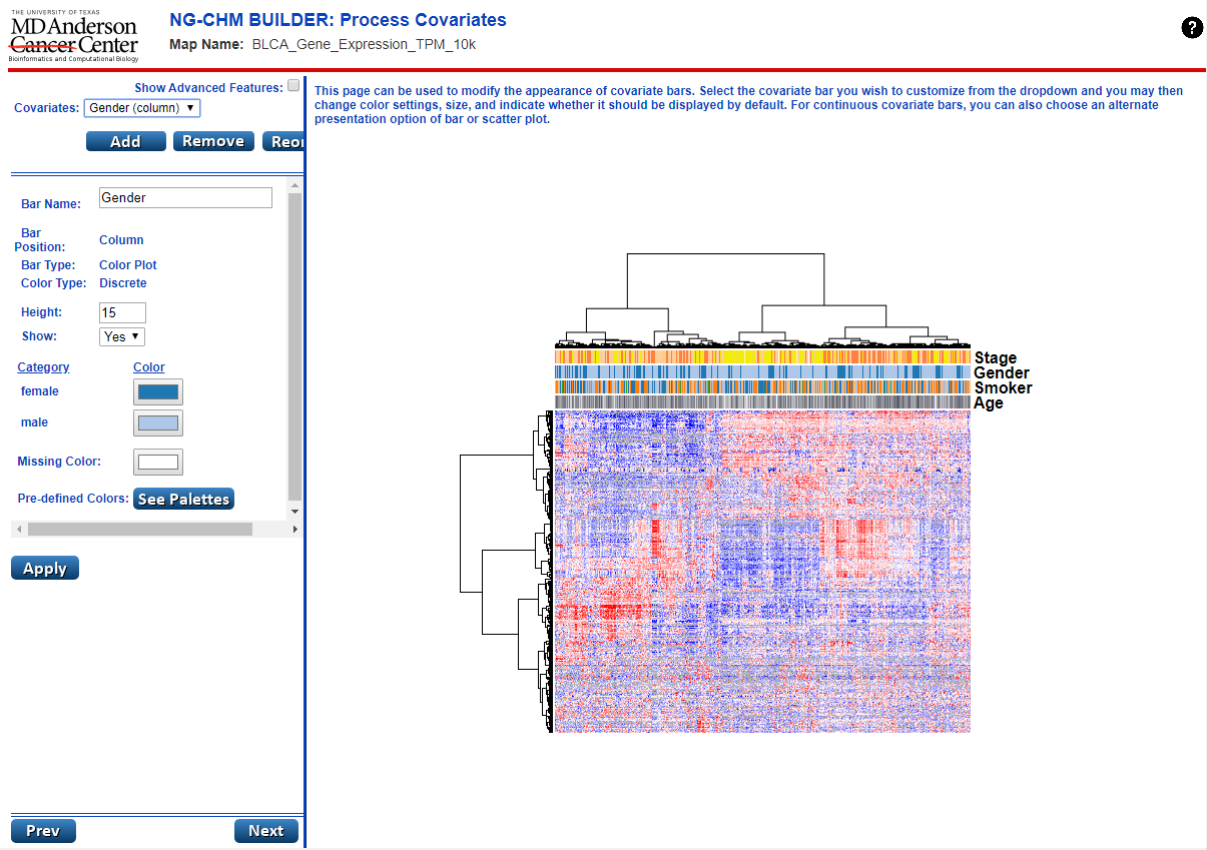
The covariate screen allows for the addition of supplemental data that describes the rows or columns of the data. This screen is also used to change the color of values and ordering of the covariate bars.

The next page allows covariate (classification) bars to be added to the heat map ( Figure 6). Covariate bars add descriptive information about the rows or columns of the heat map. A covariate bar file has the same labels as the rows or columns in the matrix and an annotation value. In this use-case we will use TCGA clinical data to add age, smoking status, gender, and tumor stage to the heat map. The covariate file contains sample ids and clinical values – one value per line. When a covariate file is added, one must identify it as a row or column covariate and specify whether it contains discrete (categorical) data or continuous values. In this case smoker status, gender, and stage are discrete column covariates, and age is a continuous column covariate.

After covariate bars have been added, the colors associated with the covariate values can be changed. If the color scheme might be useful for other maps, the palette can be saved to the server using the See Palettes button. Covariates can be reordered on the same screen.

An advanced feature, accessed on the cluster page, is the ability to generate a covariate bar based on the clustering dendrogram. If, for example there are four distinct clusters in the data and one wants to emphasize them in discussion of the heat map, a covariate that identifies the four top clusters based on the four top branches of the dendrogram can be generated.

Another notable advanced feature is the ability to include classification data in the original matrix uploaded in the first step, rather than providing individual covariate files on the covariate page. Choosing advanced features on the first page enables the user to identify covariates as well as labels and data in the uploaded matrix.

### Format heat map

**Figure 7.**
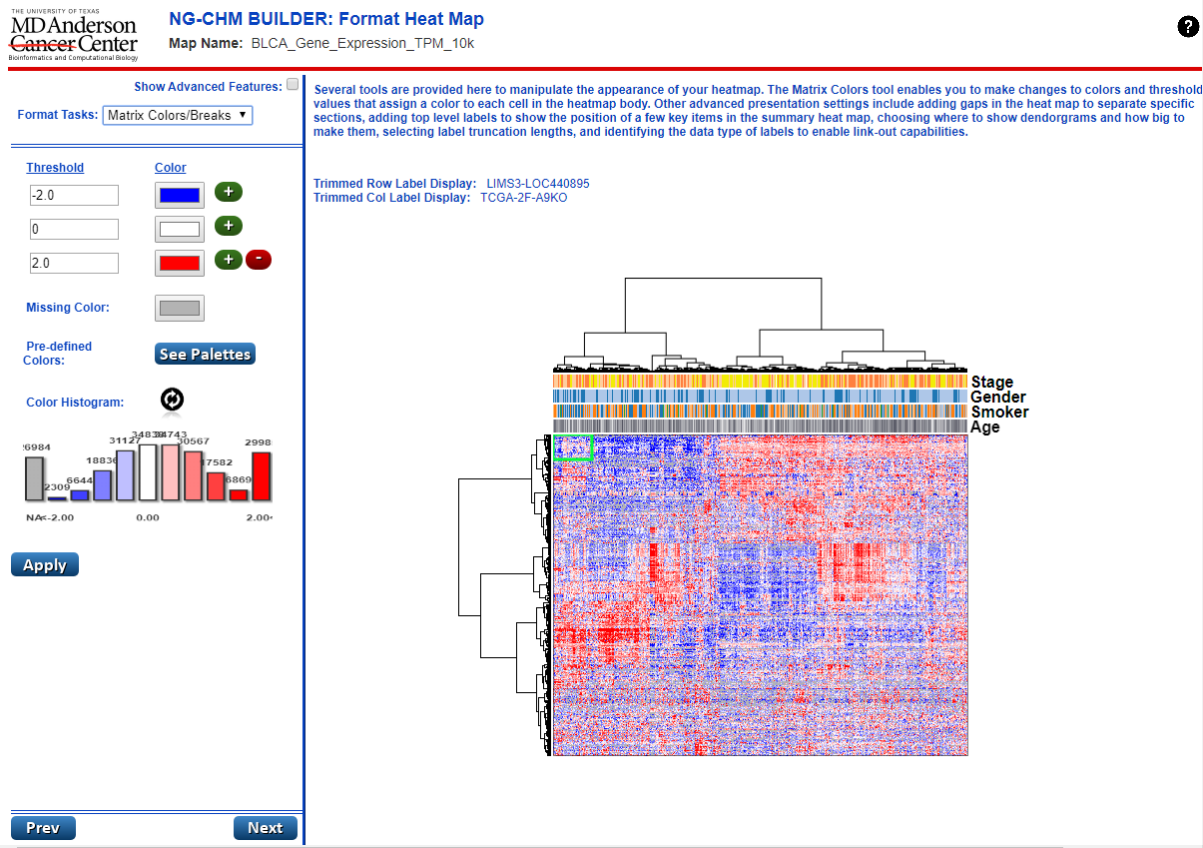
The format step is used to make changes to the appearance of the heat map, for example, changing the color scheme or altering the breakpoints associated with the colors. Many appearance change options are available.

The format screen ( Figure 7) supports the final step in generation of a heat map, adjustments of its appearance:

- Adjustment of colors and break points in the body of the heat map.
- Formatting of labels
- Formatting of the dendrograms
- Specification of the data type of the labels for link-outs.

For this use case, several changes were made: (i) a slight adjustment to the break points to emphasize high and low values in the matrix, (ii) identification of row labels as gene symbols, and (iii) identification of column labels as TCGA sample identifiers. Associating the labels with known data types activates available type-specific link-outs to external data resources.

Interesting advanced features on the same page include the addition of ‘top items’ that will be displayed in the global (i.e., full) heat map view. For example, to show the positions of a few key genes, they can be entered on the page and will show on the global heat map display. Another powerful advanced feature is the ability to add gaps to emphasize sub-groups in the heat map.

### Heat map – view and download

**Figure 8.**
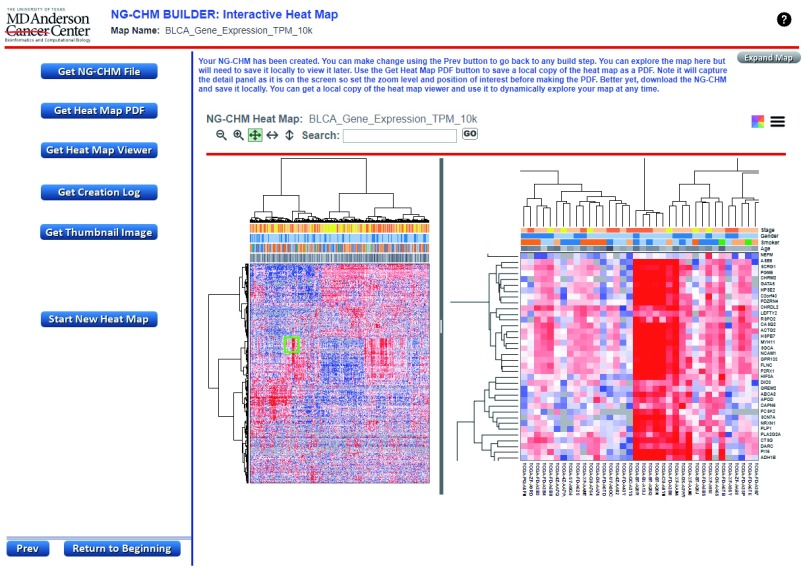
The heat map review and download screen shows the completed heat map, allows for dynamic exploration of the map, and provides download options for a PDF, an NG-CHM, and/or the construction history.

The heat map is now complete, but the Prev button can still be used to go back to previous build steps to try different options. On this final page of the Interactive Builder ( Figure 8), the map can be explored dynamically and downloaded. The Get Heat Map PDF button downloads a PDF of the summary and/or detail views as they appear on the screen – including a version of the detailed view zoomed as desired. The legends and other metadata are shown on a separate page of the pdf. The final screen can also be used to explore the dynamic heat map by zooming, panning, searching, dendrogram selection, and link outs. Clicking the Expand Map button devotes the whole browser window to the map.

Heat maps constructed on the Interactive Builder website are not saved. However, NG-CHMs can be downloaded to save and explore dynamically on your own computer. Select the Get NG-CHM file to obtain a map and then select the Get Heat Map Viewer to get a stand-alone NG-CHM viewer to run on your computer. See our NG-CHM site for more details on the capabilities of dynamic heat maps, additional builders to generate NG-CHMs (Galaxy and R) ^2^, and instructions on how to embed dynamic heat maps in your websites - https://www.ngchm.net/. Also see our YouTube channel for tutorials on NG-CHM features.

## 

### NG-CHM

The interactive NG-CHM produced by the Builder for the use case can be viewed here. Try the pan, zoom, search, and link-out features.

## Reproducibility

Reproducibility of results is becoming increasingly important for publication in high-impact journals ^14^. Therefore, it is important to be able to report the exact steps performed to transform data and create a heat map. That is particularly challenging with an iterative tool that facilitates exploration of alternative options. The Get Creation Log button on the file page of the Interactive Builder is meant to address that need. The history provided by the log shows each option, including the data transformations that were performed to produce the current map. With the original data file and the history, it is possible to recreate a heat map exactly.

## Conclusions

The Interactive CHM Builder ^9^ is an easy to use yet powerful tool for creating custom clustered heat maps for any type of study that generates a matrix of data. It has an intuitive step by step process to prepare the data and build high-quality CHMs. A sample dataset is built-in so it takes just seconds to try out the process and become familiar with the basic steps for heat map generation. It is also easy to back up to previous steps or data states to try alternative approaches and refine formatting. Finally, heat maps can be downloaded as either PDF files or NG-CHM files that support in-depth exploration of the maps.

Although there are many methods available to correct/normalize/filter data, perform hierarchical clustering, and present the resulting heat maps, most of them require programming and biostatical skills. For non-programmers the options are more limited. The best-known software packages for that purpose are Cluster 3.0 ^15^ for data manipulation and clustering combined with TreeView ^16^ for display of heat maps. Newer tools in the category include Morpheus ( https://software.broadinstitute.org/morpheus/) and Heatmapper ^17^. Some advantages of the Interactive CHM Builder are:

- Unlike Cluster 3.0/TreeView, no software installation and configuration are required. Interactive CHM Builder is available as a web service.
- Unlike other heat map tools, Interactive CHM Builder provides a step by step process starting with an unprocessed matrix that includes: correction of invalid/missing values, data normalization and transformation, data filtering, clustering, addition of covariates, and advanced customization of heat map display including link outs. At each step of the process we provide histograms and incremental heat map visualizations to assist with understanding the data and the effect of option selection.
- It is a fluid tool that supports the iterative nature of heat map creation, enabling users to move easily back and forth to revisit and modify any step of the process.
- Unlike other tools, it provides a complete history of each option selected to transform the data and generate the heat map. That capability enables the user to reproduce the heat map even months or years later.
- Finally, the resulting NG-CHMs provide enhanced ability to support dynamic exploration of patterns in the data. They can be shared with collaborators and larger research communities on a website with an NG-CHM plugin or as a stand-alone heat map and viewer.

## Data availability

Open Science Framework: NG-CHM Interactive Builder Use-Case Data. https://doi.org/10.17605/OSF.IO/H7ZS2 ^13^.

This project contains the sample TCGA bladder cancer matrix used in the use-case.

Data are available under the terms of the Creative Commons Zero "No rights reserved" data waiver (CC0 1.0 Public domain dedication).

## Software availability

**The Interactive CHM Builder is freely available for use as a web resource at:** https://build.ngchm.net/NGCHM-web-builder/. 

**Source code available from:** https://github.com/MD-Anderson-Bioinformatics/NG-CHM_GUI_BUILDER.

**Archived source code at time of publication:** https://doi.org/10.5281/zenodo.3460673 ^9^.

**License:** GNU General Public License version 2.
