# Supplementary material for: Interactive Clustered Heat Map Builder: An easy web-based tool for creating sophisticated clustered heat maps
Source: F1000Res. 2020 Mar 19;8:ISCB Comm J-1750. Originally published 2019 Oct 14. [Version 2] doi: 10.12688/f1000research.20590.2 (PMC7111501; doi:10.12688/f1000research.20590.2)
Supplement: Peer review history

# Peer review: Reviewer response for version 2

**DOI:** 10.5256/f1000research.25215.r61493

**Published:** 2020-03-30

**Authors:** Melissa S. Cline

**Affiliations:** 1 Genomics Institute, University of California, Santa Cruz, Santa Cruz, CA, USA

The authors have faithfully addressed the reviewers' feedback. This is a well-written write up of an excellent piece of research software, and represents a fine resource for the research community.

I confirm that I have read this submission and believe that I have an appropriate level of expertise to confirm that it is of an acceptable scientific standard.

# Peer review: Reviewer response for version 1

**DOI:** 10.5256/f1000research.22637.r59179

**Published:** 2020-02-21

**Authors:** Melissa S. Cline

**Affiliations:** 1 Genomics Institute, University of California, Santa Cruz, Santa Cruz, CA, USA

Ryan *et al.* present a manuscript for the interactive clustered heat map builder tool that has been widely used in cancer research consortia. The tool is of excellent quality overall, is very useful, and is intuitive in its design and execution. The manuscript is well-written, with some caveats below.

 Major feedback:

 The authors are not doing justice to the tool, which offers much more than user-friendly heatmap generation. To put the functionality in perspective, they should contrast it with the Cluster/TreeView suite, which also offers a user-friendly interface to filtering and data transformation.

 Minor feedback

 The Operation subsection assumes knowledge of Docker and Tomcat. The authors should cite appropriate background reference material for readers who aren't familiar with these technologies.

 For the use case, the authors summarized how they transformed the data, but did not indicate how those transformations were done with their tool. This needs to be clarified, because it's not obvious.

 The sample data in the OSF Storage site is stored as a single tarball. This is awkward, as the entire tarball has to be downloaded and expanded in order to access any single file.

I confirm that I have read this submission and believe that I have an appropriate level of expertise to confirm that it is of an acceptable scientific standard, however I have significant reservations, as outlined above.

# Response

**Published:** 2020-03-11

**Authors:** Michael Ryan

**Affiliations:** In Silico Solutions, USA

Thank you for your feedback and suggestions.  Below we have described how each was addressed.

***Major feedback:***

***The authors are not doing justice to the tool, which offers much more than user-friendly heatmap generation. To put the functionality in perspective, they should contrast it with the Cluster/TreeView suite, which also offers a user-friendly interface to filtering and data transformation*.**

Good suggestion.  We have modified the 5 ^th^ sentence in the first paragraph of the introduction to broaden the description of the scope of the tool and have included a new paragraph in the conclusion section to contrast our tool with Cluster 3.0/Treeview.

***Minor feedback***

***The Operation subsection assumes knowledge of Docker and Tomcat. The authors should cite appropriate background reference material for readers who aren't familiar with these technologies.***

We agree.  Additional detail including links to the appropriate reference material for Docker and Tomcat, has been added to the Operation section.

***For the use case, the authors summarized how they transformed the data, but did not indicate how those transformations were done with their tool. This needs to be clarified, because it's not obvious.***

Thank you for pointing that out.  The transforms section of the use case has been modified to provide the exact path through the screen options for each transform performed.  That should make it easier to follow the steps exactly.

***The sample data in the OSF Storage site is stored as a single tarball. This is awkward, as the entire tarball has to be downloaded and expanded in order to access any single file.***

Agreed.  Accordingly, we have modified the OSF Storage site to contain a folder with all of the files needed for the use case as individual, uncompressed files.

# Peer review: Reviewer response for version 1

**DOI:** 10.5256/f1000research.22637.r55133

**Published:** 2019-10-29

**Authors:** Natasha Caplen, Soumya Sundara Rajan

**Affiliations:** 1 Genetics Branch, Center for Cancer Research, National Cancer Institute, Bethesda, MD, USA

Ryan and co-workers have developed the software tool Interactive Clustered Heat Map (CHM) builder to enable investigators with minimal expertise in bioinformatics and biostatistics to generate publication-quality heatmaps. The use of heatmaps to visualize related datasets is a common feature in many reports of the results of studies that include genome or transcriptome-scale experiments. However, the statistical underpinnings of a heatmap require the application of appropriate transformation and clustering procedures. The interactive CHM tool makes use of user-uploaded data that is then processed to generate heatmaps defined by a set of standardized options; for example, the user can select different distance metrics (e.g., the calculation of Euclidean distance versus Manhattan distance) or clustering options (random versus hierarchical). The user also has the option to input possible co-variant data sets for the further stratification of the primary results. Furthermore, the user can customize the visual properties of the heatmap by selecting the output of the computational pipeline from a palette of colors. The article itself is well-written, though, as stated below, we recommend some edits to the current text. A particularly positive feature of this CHM tool is the inclusion of a dynamic capability that allows the user to explore their data in greater depth. Many of the features of the graphical user interface (GUI) are easy to use, and the user does not have to refer to the accompanying article describing the builder software continually. However, to enhance the impact of this resource, we recommend modification of the current versions of their article and software tool to address the following points. 

**Article**

 In the Introduction, the authors discuss the user’s ability to use their tool to generate heatmaps reiteratively, refining data transformation, annotation, clustering, and formatting. The authors also point out that this may introduce the risk of generating a multiple-comparison issue. To help the user avoid such issues, can the authors briefly mention other resources (e.g., review articles) that the user can refer to when considering which of the transformation, clustering, and distance metrics will be most applicable to their dataset?

 The authors should include a discussion of how the interactive HCM builder compares to other free heatmap generators available, for example, heatmapper.ca; Babickiet al., Heatmapper: web-enabled heat mapping for all *Nucleic Acids Res. 2016 May 17 (epub ahead of print). DOI:10.1093/nar/gkw419*); and the Morpheus software from the Broad Institute ( https://software.broadinstitute.org/morpheus/).

 Some datasets require non-hierarchical clustering to obtain the most appropriate and meaningful interpretation of the results. Please explain why this software provides only either hierarchical, random, or no clustering options?

**Website**

 Some test runs found that when the user runs through the work-flow and generates a heatmap using a dataset, the generation of a new heatmap either using the same dataset or a different dataset requires the user to close the website and re-open the homepage. The re-set function may need modification.

 Some test runs found that when choosing the formatting and then palettes after adding co-variants, the apply button on the left-hand window has lines running through it.

 It is easy to maneuver and resize the highlighter box over any region of the heatmap generated using the sample data. However, we noted not all heatmaps performed as well using user-uploaded data.

 Please state clearly on the website’s front-page that the website limits the heat map to “no more than 4,000 total rows and columns and no more than 3,500 elements on either axis.” In the absence of this statement on the front-page, users may attempt to upload more complex datasets.

We confirm that we have read this submission and believe that we have an appropriate level of expertise to confirm that it is of an acceptable scientific standard.

# Response

**Published:** 2020-03-11

**Authors:** Michael Ryan

**Affiliations:** In Silico Solutions, USA

Thank you for your detailed comments and suggestions on the article and the tool.  Each suggestion/comment is addressed below:

***Article***

******

***In the Introduction, the authors discuss the user’s ability to use their tool to generate heatmaps reiteratively, refining data transformation, annotation, clustering, and formatting. The authors also point out that this may introduce the risk of generating a multiple-comparison issue. To help the user avoid such issues, can the authors briefly mention other resources (e.g., review articles) that the user can refer to when considering which of the transformation, clustering, and distance metrics will be most applicable to their dataset?***

We are not aware of any review article that covers the topic comprehensively.  But, in the text, we do cite an article of ours (Weinstein JN: A postgenomic visual icon. Science. 2008;319: 1772) that provides additional background on some of the relevant subtleties of heat map generation.  As you correctly point out, the optimum approach depends on the specifics of an individual dataset and objectives of the study.  In light of your comment, we will consider writing a review article that addresses those issues at more length.  Also, we are contemplating a future enhancement to the Interactive CHM Builder that would provide templates or workflows based on study type as a starting point to assist with navigating data transformations and heat map generation.

***The authors should include a discussion of how the interactive HCM builder compares to other free heatmap generators available, for example, heatmapper.ca; Babickiet al., Heatmapper: web-enabled heat mapping for all*** ***Nucleic Acids Res. 2016 May 17 (epub ahead of print). DOI:10.1093/nar/gkw419*** ***); and the Morpheus software from the Broad Institute (*** ***https://software.broadinstitute.org/morpheus/*** ***).***

Thank you for the suggestion.  We have added a paragraph to the conclusions section to enumerate what we feel are the advantages of our Interactive CHM builder compared with the other similar tools.

***Some datasets require non-hierarchical clustering to obtain the most appropriate and meaningful interpretation of the results. Please explain why this software provides only either hierarchical, random, or no clustering options?***

We agree.  Thank you. The methods we have currently implemented are the ones that are most heavily used in publications of omics research.  We will add non-hierarchical clustering methods to our requested features list for a future release.

***Website***

******

***Some test runs found that when the user runs through the work-flow and generates a heatmap using a dataset, the generation of a new heatmap either using the same dataset or a different dataset requires the user to close the website and re-open the homepage. The re-set function may need modification.***

Thank you for reporting this issue.  We have modified the restart flow and believe the problem has been corrected.

***Some test runs found that when choosing the formatting and then palettes after adding co-variants, the apply button on the left-hand window has lines running through it.***

We have been unable to reproduce that issue in the latest release of the software so we believe it has been corrected.  If you encounter it again, we would appreciate it if you submit a git issue, noting the browser and operating system for which it occurs.

***It is easy to maneuver and resize the highlighter box over any region of the heatmap generated using the sample data. However, we noted not all heatmaps performed as well using user-uploaded data.***

Thank you for the report.  Since submission of the paper, we have made several improvements to the selection/sizing features and have tested many odd sized asymmetrical matrices.  We will continue to implement improvements in selection mechanics if additional issues arise.

***Please state clearly on the website’s front-page that the website limits the heat map to “no more than 4,000 total rows and columns and no more than 3,500 elements on either axis.” In the absence of this statement on the front-page, users may attempt to upload more complex datasets.***

For many studies, an important step in preparing data for clustering and heat map generation is filtering out rows and/or columns that have a high proportion of missing values or that show little variance across samples.   We want to allow users to upload matrices that are above the clustering limit because the filtering step will often reduce the size of the matrices such that they can be clustered.  The manuscript was not clear on this point.  Thank you for pointing this out.  We have modified the Use Case section “Transform/filter the data”, paragraph 2, to explicitly discuss clustering limits and the use of filtering to reduce larger datasets.

The interactive nature of the tool does limit the maximum matrix we can cluster.  As you know, the compute time for most clustering algorithms essentially increases as the square of the largest dimension.  The tool’s limit for the clustering step has been increased from 4,000 to 5,000 total rows/columns. The 3,500 axis limit has been removed.  We’ve also added new system messages that more clearly explain those issues, and we plan to continue pursuing increases in the limits as computational power increases and clustering algorithms advance.
